# An alternative reference space for H&E color normalization

**DOI:** 10.1371/journal.pone.0174489

**Published:** 2017-03-29

**Authors:** Mark D. Zarella, Chan Yeoh, David E. Breen, Fernando U. Garcia

**Affiliations:** 1 Department of Pathology & Laboratory Medicine, Drexel University, Philadelphia, PA, United States of America; 2 Department of Electrical & Computer Engineering, Drexel University, Philadelphia, PA, United States of America; 3 Department of Computer Science, Drexel University, Philadelphia, PA, United States of America; 4 Department of Pathology & Laboratory Medicine, Cancer Treatment Centers of America, Eastern Regional Medical Center, Philadelphia, PA, United States of America; Advanced Centre for Treatment Research and Education in Cancer, INDIA

## Abstract

Digital imaging of H&E stained slides has enabled the application of image processing to support pathology workflows. Potential applications include computer-aided diagnostics, advanced quantification tools, and innovative visualization platforms. However, the intrinsic variability of biological tissue and the vast differences in tissue preparation protocols often lead to significant image variability that can hamper the effectiveness of these computational tools. We developed an alternative representation for H&E images that operates within a space that is more amenable to many of these image processing tools. The algorithm to derive this representation operates by exploiting the correlation between color and the spatial properties of the biological structures present in most H&E images. In this way, images are transformed into a structure-centric space in which images are segregated into tissue structure channels. We demonstrate that this framework can be extended to achieve color normalization, effectively reducing inter-slide variability.

## Introduction

Hematoxylin and Eosin (H&E) staining variability is ubiquitous in pathology, and has significant consequences on the digital pathology workflow. Image analytics that can potentially serve an important role in computer-aided diagnostics may become compromised in the presence of high variability. In particular, algorithms that rely on pixel color or intensity to characterize tissue in an automated fashion (e.g. [1–5]) may benefit from increased color homogeneity in the image samples. Color standardization therefore becomes an important preprocessing step for many computational algorithms [6]. Likewise, methods to ensure laboratory compliance with staining protocols should be developed to ensure high quality patient care [7].

An improved method for H&E staining standardization and calibration can reduce the obstacles presented by staining variability. Recent work has used color normalization as a tool to transform H&E images to conform to a standardized color target in a manner that minimizes the loss of useful information in the image. Many of these approaches have used intensity thresholding [8, 9], histogram normalization [10], stain separation [11–13], and color deconvolution [14–16] to characterize or normalize H&E images, with varied levels of success. These algorithms shared a common strategy in that normalization can be approached from the standpoint of color analysis: that the superposition of stains that form the color basis of an image can be deconstructed into its constituents and recombined to conform to a target. A recent test [17] of two state-of-the-art algorithms demonstrated that color normalization can reduce the significant color variation across images by a factor of nearly 2, while retaining most of the intra-image pixel variance that may correspond to useful image details. Likewise, Bautista et al. [9] recently showed that the dissimilarity in color that resulted from differences in whole slide scanners could be reduced by a factor of 3–4 using a color calibration technique. However, even with advanced normalization tools, residual variability across images persists; in the data set used by Sethi, et al. [17], this residual inter-image variability after color normalization amounted to approximately 10% of the total range (estimated by the standard deviation that they reported). Given that the differences in the color properties of some important histological structures may often be even less than 10% [18, 19], improvements to reduce inter-image variability must therefore still be made in order to consistently and reliably distinguish between histological structures and interpret the tissue correctly.

In this paper we present an algorithm to achieve the same function as previous color normalization algorithms, in that a transformation of the color attributes of the image can be applied to imitate a standardized color distribution. However, our algorithm accomplishes this function via an analytical process that explicitly relates colors to histologic structures, thereby harnessing the spatial information in the image to guide color normalization and improve performance.

## Materials and methods

### Color reduction

Central to our algorithm is the hypothesis that pixels that share similar colors have a high likelihood of belonging to similar structures. We have previously noted [18] that reducing H&E images to ten colors in breast cancer images did not visibly result in a substantial loss of detail. Here we quantified this observation by measuring the ratio of intra-cluster to inter-cluster Euclidean distances as a means to describe the color relationship between pixels. Guided by these results, we reduced each 8-bit source image to ten colors using k-means clustering. We converted the image to HSV space, where each pixel represents hue, saturation, and value in three channels. Since HSV is a cylindrical coordinate system, we performed clustering by first transforming pixel values into a Cartesian coordinate system and then using each dimension as a separate variable for clustering. As a result, k-means clustering sought to minimize Euclidean distances between pixels belonging to the same cluster. We mapped each pixel in a cluster to the HSV value of the cluster’s centroid, establishing a ten-color image.

### Color assignment

We manually classified each of the ten colors into one of the following categories: white/lumen, stroma, nuclei, and cytoplasm, in the same manner as previously reported [18]. We used an interactive user interface developed in Matlab (Mathworks, Natick, MA) to perform this task, whereby an individual color could be highlighted in the H&E image to emphasize to the user the spatial distribution of that color in the image. Each color was classified by the user when it became apparent that it belonged to a single tissue element. For instance, if highlighting a color revealed that it belonged solely to nuclei, the user would associate it with “nucleus” in the interface. Colors that belonged to a mixture of tissue elements (e.g. a single color shared by cytoplasm and nuclei) were left unassigned. In many cases, a tissue element was represented by multiple colors; users selected all colors that belonged to that tissue element to ensure optimal color representation.

### Pixel classification

Given that some colors may not have been assigned to one of the four tissue element groups, we applied a second classification step to ensure that all pixels in an image were classified. We used the color assignment step described above to serve as the ground truth for a support vector machine- (SVM-)based learning algorithm that partitioned HSV space into four regions. We used a linear kernel and a soft margin box constraint of 1. In order to accommodate four different classes, we embedded the SVM classifiers within a decision tree framework that first delineated pixels belonging to white space from pixels belonging to tissue, then stroma from putative epithelial tissue, and finally nuclei from cytoplasm. We considered the certainty of the classification for a given pixel to be proportional to the distance between the pixel’s HSV coordinates and the hyperplane that determined its class. The result of this analysis is a four channel data structure in which each channel represents a structure of interest. Negative values indicate a low likelihood that the pixel belongs to a particular class. A class map can be generated by applying the absolute value to this data structure. We modulated class maps by certainty values for visualization purposes.

### Color normalization

We developed a procedure to map pixels to a target color that was assigned to each of the four tissue elements. We used a test image set to derive a set of target colors by measuring the average colors associated with white space, stroma, nuclei, and cytoplasm, though this designation was arbitrary and did not significantly impact the results reported here. We mapped a classified image to the target using a three step process: 1) measure the deviation, Δd, of each pixel about the measured mean color (in HSV coordinates) of each tissue element in the image; 2) remap every pixel in the image to its target color based on its classification, and modulate the pixel about the target color by Δd; 3) constrain the resultant values to [0,1]. This procedure produced a color normalized image based on the structure classification provided by the user.

### Case selection and testing

We analyzed 58 breast cancer images provided as a public repository by the UCSB Center for Bio-image Informatics. This image set was accompanied by manual annotations of the nuclei with the intended purpose of nuclear segmentation algorithm performance evaluation [20]. For comparison, we also included analysis of one image from the Drexel University College of Medicine breast cancer databank. All images were de-identified.

To measure the inter-image reduction in color variability that resulted from color normalization, we measured the standard deviation of the mean pixel values in HSV space for each tissue structure before and after normalization. This analysis provided quantitative insight into the amount of color variability present in the original images and the variability that persisted after normalization, and has previously been used in other reports [17]. Despite the pursuit to significantly reduce unwanted inter-image variability, an ideal for most color normalization algorithms is that the inherent variability within images is preserved. We measured the intra-image standard deviation to demonstrate the persistence of variability after normalization by computing the variability of pixel color within each tissue structure in a given image. We used the Wilcoxon Sign Rank test to test the null hypothesis that the distribution of intra-image standard deviations across the data set was not changed by color normalization.

To quantify the amount of potentially meaningful variability retained after color normalization, we measured the mutual information between the unnormalized (X) and normalized (Y) images. We expressed this value as the fraction of an optimal value by dividing the measured mutual information by the mutual information between the unnormalized image and itself, generating the normalized mutual information (NMI).

$$NMI=\frac{MI(X,Y)}{MI(X,X)}$$ (1)

To demonstrate that classification alone, without the scalar structure associated with color normalization, cannot preserve this information we computed the NMI for the original image and the output of the normalization algorithm without the pixel-level modulation described by Δd in the previous section.

To evaluate the impact of noise on NMI, we added Gaussian random noise to unnormalized images and computed the NMI between the original image and noise-added image. Noise was added to the image in HSV space isotropically at varying σ values to control the amount of noise added (σ = 0.001, 0.005, 0.01, 0.05, 0.1, 0.5). For this analysis, NMI was measured in the value channel and the hue-saturation plane.

## Results

We developed a multi-stage algorithm, designed to operate with a minimal number of free parameters, to rapidly transform H&E images into a space more amenable to image processing and quantification with a primary goal of mitigating staining variability across images. This processing framework is divided into four main steps: 1) clustering by color to reduce the complexity of the image into a tractable set of channels; 2) assigning individual color channels to tissue elements to establish anchor points for calibration; 3) applying machine learning to parse the entire image into the assigned tissue elements; 4) transforming the classified image into a standardized space. This standardized space can include, for example, a nuclear likelihood space to aid in nuclear segmentation. However, we approach this report from the standpoint of color normalization, and therefore the standardized space we focus on is defined by a target that will enable more homogeneous visualization across images.

### Color reduction

**Fig 1:**
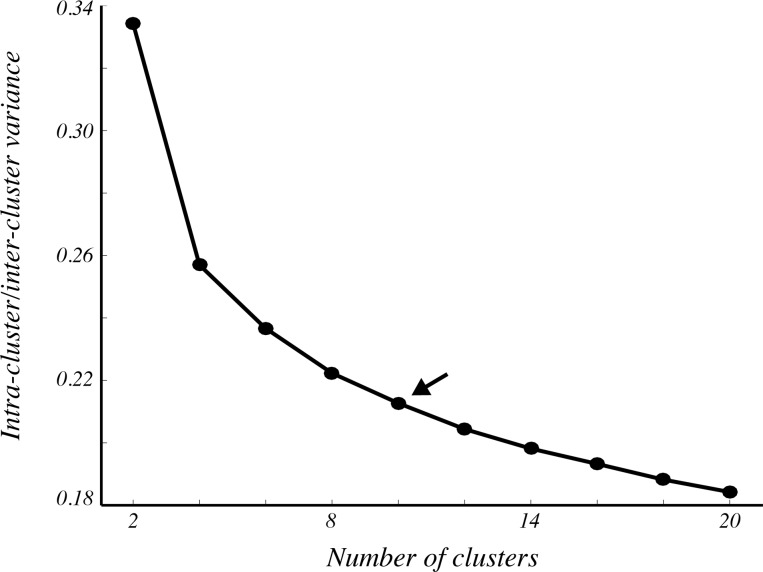
Color dissimilarity decreases with number of clusters. The ratio of intra-cluster to inter-cluster variance decreased in H&E images as pixels were divided into more clusters. Each point represents the mean intra-/inter-cluster ratio averaged across all clusters and all 58 cases. The arrow denotes ten clusters, which we used for the remainder of the analyses.

The algorithm begins with the reduction of the complex color attributes of the image down to a tractable set of colors that can be individually characterized based on their spatial distribution in the image. As we demonstrated previously in breast cancer images [18], reduction of the color depth of H&E images from approximately 256 colors to 10 colors preserves most of the information important for identification of biological structures such as nuclei, cytoplasm, and stroma. To confirm that a 10 color representation adequately captures much of the color variability in our image set, we measured intra-class color variance following k-means clustering. By normalizing intra-class variance by inter-class variance, we measured the distinctness of pixel colors within orthogonal color channels as a surrogate measure of color information retained by clustering. Predictably, we found that the normalized variance of H&E images decreased as the number of clusters was increased (Fig 1). It also became apparent that the rate of this decrease slowed as the number of clusters increased. For instance, the reduction of normalized intra-class variance was less than 0.04 when comparing 10 colors to 20 colors, implying that the training complexity associated with using more than 10 colors may yield negligible improvements in accuracy. We chose 10 clusters for the remainder of our analyses.

### Pixel classification

Using the spatial distribution of the colors that comprise an H&E image and their association with tissue elements, we manually segregated a subset of the ten clustered colors into four categories: white space, nuclei, cytoplasm, and stroma, while potentially leaving other colors unassigned. In order to classify all pixels in the image (including those that were not segregated by k-means in a way that retained their association with a structure), we used machine learning to divide color space into four unambiguous regions. We embedded support vector machine (SVM) learning [21] within a decision tree framework in such a way that binary classification occurred in a stepwise manner based on color similarity. First, white space was discriminated from tissue; then, stroma was discriminated from epithelial tissue; then nuclei and cytoplasm were distinguished from one another. In this way, HSV space was partitioned into regions in which colors defined structures, and this segregation was specific to the image being processed. The data labels that trained the classifiers were derived from spatial properties rather than absolute colors, therefore being immune to inter-slide staining variability.

**Fig 2:**
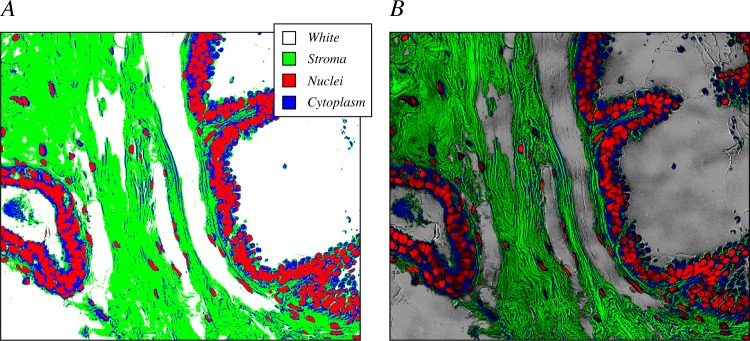
Maps of classification accompanied by a measure of certainty preserve image detail. (A) An example binary classification map shows that pixels are designated as nuclei (red), cytoplasm (blue), stroma (green), or lumen/white space (white). (B) Modulation of the binary classification map in (A) by the normalized distance from the classification hyperplane introduces a scalar value that restores detail to the image, and can be used as a measure of classification certainty for each pixel.

This classification step resulted in a map in which each pixel was assigned to one tissue element (Fig 2A). This map, however, lacks much of the spatial detail that distinguishes between element boundaries, gradations, and nuance present in the original H&E images. Therefore, the utility of this representation is fundamentally limited. For example, it is impossible to accurately segment individual nuclei from the larger structures that they form, and it is difficult to discern stromal patterns and their variability across space. However, when the classification map was modulated by the distance of the pixel from the classification hyperplane, which serves as a measure of classification certainty, much of the detail that was lost re-emerged (e.g. Fig 2B). With this representation, it becomes easier to distinguish subtle patterns that aid in segmentation, for example. Furthermore, it allows pixels that are not strongly classified to be identified, enabling additional processing steps to exclude them from some analyses.

### Color normalization

By projecting H&E images into a structure-centric framework, the color properties of the image are aligned to anchor points defined by common structural elements that are shared among H&E images typically under study. The steps up to this point have effectively transformed an image that is modulated by color variation, which lacks an intrinsic relevance, to one that is modulated by classification certainty. This representation is useful on its own; for example, nuclear segmentation can be performed by operating on the nuclear channel of the new framework rather than on a grayscale intensity image. However, color normalization is achieved by defining a set of target colors that represent structures of interest in a standardized manner, and modulating the pixel values in an image around those colors. For the purposes of this study, we selected the mean color values of each structure across all 58 test images as the target colors.

**Fig 3:**
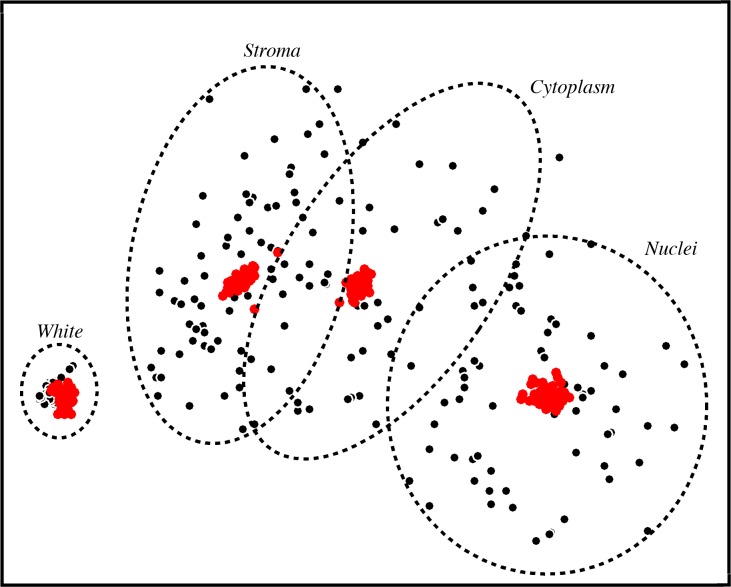
Color representation overlap is substantially reduced following color normalization. The mean HSV value for each designated structure was computed for all 58 cases and projected to two dimensions for visualization purposes using multidimensional scaling. Black points represent the raw uncorrected images; red points represent the same images after color normalization. Circles denote the vicinity of the structures and illustrate that substantial overlap exists in the unnormalized image centroids but disappears after normalization.

**Fig 4:**
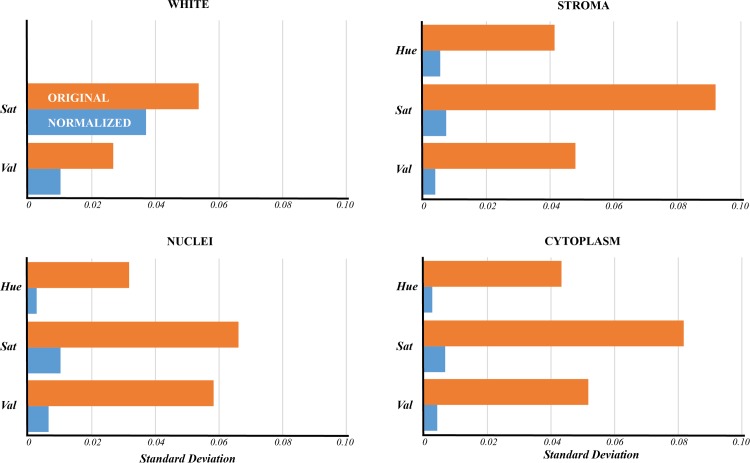
Color normalization reduces variability within the hue, saturation, and value channels. Color variability following normalization was measured as the standard deviation of pixel values in HSV space across all four tissue classes (blue bars). When compared with the same measure in the unnormalized images (orange bars), a significant reduction was observed (Wilcoxon sign rank test, *p*<0.01). Hue was left out of the analysis for white space because the color white does not contain a meaningful hue, as it has near-zero saturation.

In Fig 3, we show the variability of colors, with an approximation of the separation by tissue element, for all 58 test images in a two-dimensional projection of HSV space derived using multidimensional scaling. Each black point represents the mean color of one structure in a given image. The considerable variation observed reflects the significant color variability that exists in this image set, and results in overlap between structures that is difficult, if not impossible, to consistently segregate by color across all images. However, after applying the color normalization algorithm, a significant reduction in the variability across images is achieved (Fig 3, red points). The inter-image variability of the unprocessed images (Fig 4, orange bars) was substantially greater than after color normalization (Fig 4, blue bars) for stroma, nuclei, and cytoplasm (a reduction by a factor of 6–16). White space variability was reduced by a lesser factor (approximately 2) for saturation and value. Hue variability was not analyzed for white space, as hue is not a meaningful quantity for the color white.

**Fig 5:**
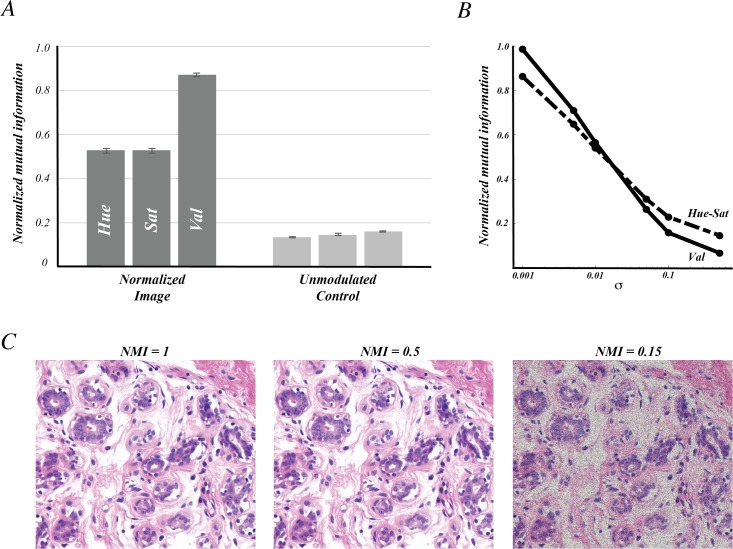
Modulation by individual pixel values preserves image detail. (A) The normalized mutual information (NMI) refers to the ratio of the mutual information between normalized and unnormalized images and the mutual information between the unnormalized image and itself. This ratio was measured for each color channel individually (dark bars). As a control, the same metric was applied to compare an image projected to the same normalized space without modulation about the mean to the unnormalized image. In this way, only the pixel’s class (e.g. nuclei, stroma) contributed to the prediction of the original image. (B) The NMI was computed for noise-added images and its value depended on the amount of noise added, represented by σ. Measurements were made separately along the Value channel (Val) and Hue-saturation plane (Hue-Sat). (C) Representative unnormalized images for one case are shown after different amounts of noise were added corresponding to *NMI = 1* (left), *NMI = 0*.*5* (middle), and *NMI = 0*.*15* (right). For visualization purposes, images were saturated to account for pixel values out of range. This did not contribute to the measurements shown in (A) or (B).

Notably, a similar reduction in variability can be achieved by simply classifying the tissue structures in the image, without modulating the pixels according to the properties of the original image. However, as shown in Fig 2, scalar information that accompanies the classified image can provide substantial detail that has utility in many image analysis paradigms, and certainly improves visual interpretation of the image. We introduced a scalar component to the classified image by measuring the color deviation of pixels about the mean for each tissue element. We found that this operation resulted in structure-specific intra-image variability not significantly different from the unnormalized image, and similar to that reported previously [17]. To test how well this residual variability reflects the original modulation of the image, we measured the normalized mutual information (NMI) between the normalized and unnormalized images in HSV space (Fig 5A, dark bars). We found that the hue and saturation channels exhibited NMI values of about 0.5, while the value channel had an NMI value over 0.9, indicating very high concordance between the intensities of the two images. In contrast, by not introducing this scalar component, only moderate mutual information resulted (Fig 5A, light bars).

To aid in the interpretation of these NMI values, we measured the NMI for synthetic images constructed by adding Gaussian noise to the original unnormalized images. In Fig 5B, the NMI values obtained using σ values spanning 0.001 to 0.5 are shown, demonstrating a decline in NMI as greater levels of noise is added. The slightly sharper decline in the value channel is likely due to the fact that the variability of pixel values in the test images is lower than in the hue and saturation channels. We selected σ values that produced NMI values of 0.5 and 0.15, consistent with the NMI values obtained after color normalization and after classification, respectively. In Fig 5C, we show that there is an imperceptible difference between the *NMI = 0*.*5* noise image and the original, demonstrating that only slight modulation of the image produces an NMI of 0.5. On the other hand, the noise properties of the *NMI = 0*.*15* image are visually prominent. These results imply that the impact of color normalization on the information content of the image is likely minor.

**Fig 6:**
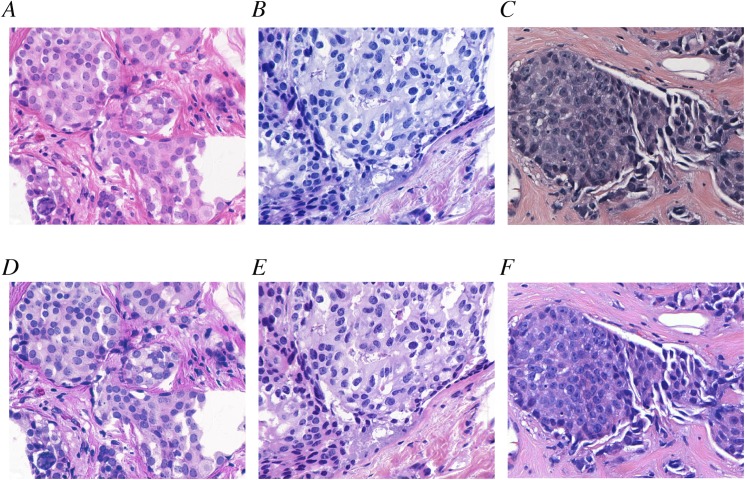
Color normalization for visualization. (A,B) Two images from the UCSB data set exhibited different staining properties. A strong red hue was observed in (A) while the image in (B) had a greater proportion of blue. (C) Likewise, a representative image from the Drexel breast cancer databank exhibited yet a different color property. (D-F) Color normalization produced visually similar color properties.

In summary, after applying color normalization, H&E images with vastly different color properties could be made to appear similar. Three such examples are shown in Fig 6. Fig 6A and 6B depict two original unprocessed images from the UCSB data set that represented samples with different color attributes. Fig 6D and 6E show the same images after color normalization was applied. Fig 6C is an example selected from the Drexel breast cancer databank with yet different color properties; Fig 6F demonstrates that color normalization using the target derived from the UCSB data set can generate an image with similar color attributes to the normalized UCSB images.

## Discussion

H&E staining variability poses a problem that is difficult to overcome in many applications. The sources of variability are present in nearly every step of the preparation and also include the inherent biological diversity of tissues [19]. To reduce variability, standardization at a number of different stages should be implemented, potentially impacting tissue preparation techniques, staining protocols, microscopy standards, and the digitization process. However, retrospective data analysis does not benefit from any of these proposed improvements, and recalibration to new standards would become necessary every time a change is adopted. We have developed an image processing approach to achieving color standardization by utilizing the rich spatial information in H&E images. This approach provides a computationally realizable solution to standardization, accommodates retrospective data analysis, and is adaptable to new protocols.

The procedure we present relies only on two free parameters: the number of clusters used for color reduction and the SVM tuning parameters. The selection of number of clusters balances accuracy with the amount of user intervention required. However, in our view, if any value within a reasonable range for this parameter is selected, the impact on the result will be relatively small. As we showed in Fig 1, for representations beyond approximately 10 colors, the reduction in intra-/inter-cluster variance begins to plateau, indicating that the natural divisions in color that tend to exist in H&E images are most prominent after relatively few clusters are selected. We cannot, however, exclude the possibility that other tissue types may exhibit more complex color attributes that require more color clusters. For instance, the parotid gland has an epithelial component with specialized cytoplasm rich in shades of blue that may need to be represented by multiple clusters to distinguish it from other structures. The framework we present here enables users to select the most appropriate number of clusters tailored to the data set under study.

Here we present an approach for combining shape analysis with color for the purposes of color representation. We exploited the correlation between the spatial distribution of pixels and their colors in order to establish a color invariant representation of H&E images. We envision this technique being augmented to include other tissue elements when other structures must be discriminated and represented. For example, lymphocytes, blood, and dyes/markings are often encountered in H&E slides, and typically come with their own color properties that likely can be accounted for using this method due to their stereotypical spatial attributes. We advocate the approach of combining spatial analysis with color analysis to include additional structures of interest.

Manual user intervention was necessary in the algorithm as we describe it. However, we expect that the accuracy of the algorithm would not suffer if automated shape-based structure classification methods were used, presuming those methods were themselves accurate. Several automated structure assignment algorithms [3, 22–29] have been developed that, in combination with the algorithm we describe, could produce a completely automated color normalization algorithm. Furthermore, given that the color assignment stage is followed by a separate classification step, there is some buffer to keep the algorithm robust in the presence of minor color assignment errors that may accompany automated classification. As noted earlier, SVM tuning parameters are one of two free parameters in this algorithm, and these especially can be tweaked to allow more accurate model generation when the ground truth is expected to have some probability of error [30].

An important requirement for the implementation of this algorithm is that the training data from which the tissue structure classifiers are derived must be representative of the data set to which it will be applied. The image scale must also be high enough to avoid color mixing artifacts that arise from inadequate resolution. The classification algorithm relies on correctly associating colors with structures; resolution, therefore, must be sufficient to resolve the tissue structures that serve as anchor points for color transformation. Using fine-scale features such as cell nuclei may require magnification of 10x or higher to adequately capture the colors associated with these structures. Future research is needed to model the aliasing effects that occur at lower resolutions from the standpoint of color in order to enable transformations to low resolution images.

Recent analysis [17] of two high performing color normalization algorithms demonstrated that reduction in color variability can be achieved using color normalization. These authors showed that images with color variability similar in magnitude to those used in our analysis could be transformed in a manner that reduced color variability by a factor of about 2. Notably, one algorithm predominantly showed this reduction in the hue channel, while the other exhibited the reduction in the saturation and value channels. In contrast, our results indicate that the algorithm we propose reduces variability in all three color channels by a factor of 6–16, a substantial improvement over current algorithms.

The amount of information preserved after normalization is an important parameter that defines the quality of the algorithm. Sethi, et al. [17] used standard deviation to describe intra-image variability and demonstrated that the Khan, et al. [16] and Vahadane et al. [11] algorithms exhibited variability similar in magnitude to the original image’s variability, concluding that much of the information in the original images was not lost. We noted a similar property in our results. However, this metric does not directly measure the ability of intra-image variability to capture useful modulation in the image. We used mutual information as a direct measure of this factor and, although difficult to compare with previous studies that did not use this measure, we believe that the results indicate that our algorithm preserves a substantial amount of the variability present in the original image. Given the several-fold decrease we observed in variability across the data set after normalization, we believe that future improvements in color normalization should be focused on continuing to improve the mutual information between the normalized and unnormalized images in order to maximize the amount of information that can be harnessed in normalized H&E images.

As new digital image analysis capabilities in pathology continue to emerge alongside the advent of whole slide imaging for diagnostics, the need for color standardization grows. The work we present serves as an essential first step toward integrating data sets collected from different laboratories. We believe that color normalization not only provides a starting point for laboratory calibration, but also aids discovery by making image analysis and machine learning algorithms more generalizable across data sets. The extent to which the performance of these algorithms is affected will vary considerably based on several factors. Naturally, an algorithm that relies on absolute measures of color or intensity will inherit the staining bias and variability of an uncorrected set of images, which may be quite large especially for multi-institutional data sets. However, the potential loss of information inherent with color normalization may be unattractive in other applications. We believe that our work provides a quantitative reference frame for the potential reduction in staining variability, which users can reference to assess its usefulness for their specific application.
